# Sex X Time Interactions in Lp(a) and LDL-C Response to Evolocumab

**DOI:** 10.3390/biomedicines11123271

**Published:** 2023-12-11

**Authors:** Federica Fogacci, Serra İlayda Yerlitaş, Marina Giovannini, Gökmen Zararsız, Paolo Lido, Claudio Borghi, Arrigo F. G. Cicero

**Affiliations:** 1 Hypertension and Cardiovascular Risk Research Center, Medical and Surgical Sciences Department, Alma Mater Studiorum University of Bologna, 40100 Bologna, Italy; federica.fogacci@studio.unibo.it (F.F.); marina.giovannini3@unibo.it (M.G.); claudio.borghi@unibo.it (C.B.); 2 Department of Biostatistics, Erciyes University School of Medicine, 38039 Kayseri, Turkey; ilaydayerlitas340@gmail.com (S.İ.Y.); gokmen.zararsiz@gmail.com (G.Z.); 3 Drug Application and Research Center (ERFARMA), Erciyes University, 38280 Kayseri, Turkey; 4 Italian Medicines Agency (AIFA), 00187 Rome, Italy; paulshore@virgilio.it; 5 Unit of Cardiovascular Internal Medicine, Department of Cardiac, Thoracic, Vascular Pathology, IRCCS Azienda Ospedaliero-Universitaria di Bologna, 40100 Bologna, Italy

**Keywords:** lipoprotein(a), PCSK9, PCSK9 inhibitor, evolocumab, women’s health

## Abstract

The aim of this study was to evaluate whether there were significant sex x time interactions in lipoprotein(a) (Lp(a)) and low-density lipoprotein cholesterol (LDL-C) response to treatment with the Proprotein Convertase Subtilisin/Kexin type 9 inhibitor (PCSK9i) Evolocumab, in a real-life clinical setting. For this purpose, we pooled data from 176 outpatients (Men: 93; Women: 83) clinically evaluated at baseline and every six months after starting Evolocumab. Individuals who had been on PCSK9i for less than 30 months and nonadherent patients were excluded from the analysis. Over time, absolute values of Lp(a) plasma concentrations significantly decreased in the entire cohort (*p*-value < 0.001) and by sex (*p*-value < 0.001 in men and *p*-value = 0.002 in and women). However, there were no sex-related significant differences. Absolute plasma concentrations of LDL-C significantly decreased over time in the entire cohort and by sex (*p*-value < 0.001 always), with greater improvements in men compared to women. The sex x time interaction was statistically significant in LDL-C (all *p*-values < 0.05), while absolute changes in Lp(a) were not influenced by either sex or time (all *p*-value > 0.05). Our data partially reinforce the presence of differences in response to treatment to PCSK9i between men and women and are essential to gain a better understanding of the relationship between LDL-C and Lp(a) lowering in response to PCSK9i. Further research will clarify whether these sex-related significant differences translate into a meaningful difference in the long-term risk of ASCVD.

## 1. Introduction

The International guidelines for the prevention of atherosclerotic cardiovascular disease (ASCVD) recommend the use of proprotein convertase subtilisin/kexin type 9 (PCSK9) inhibitors (PCSK9i) in high-risk patients as second-line lipid-lowering agents in addition to the maximally tolerated statin dose [1]. A comprehensive meta-analysis of phase II and phase III clinical trials evaluating the effect of PCSK9i Evolocumab and Alirocumab on lipoprotein(a) (Lp(a)) concentration concluded significant improvements from baseline according to the comparator group (placebo: mean difference (MD): −27.9%, 95% confidence interval (CI): −31.1% to −24.6% versus ezetimibe: MD: −22.2%, 95% CI: −27.2% to −17.2, *p*-value: 0.04) and duration of treatment (≤12 weeks: MD: −30.9%, 95% CI: −34.7% to −27.1% versus >12 weeks: MD: −21.9%, 95% CI: −25.2% to −18.6%, *p*-value < 0.01) [2]. More recently, the small interfering ribonucleic acid (siRNA) Inclisiran has been shown to lower Lp(a) by an average of −20.9% (95% CI: −25.8% to −15.99%) [3], although the interindividual variation following treatment appears high [4].

In the last decade, substantial evidence from epidemiological and experimental studies clearly showed that high levels of Lp(a) are an independent and genetically determined risk factor for the development of atherosclerosis and ASCVD, such as coronary artery disease (CAD), stroke and aortic stenosis [5,6]. Thus, the absence of available therapeutic options for effectively managing patients with hyperlipoproteinemia(a) means that identifying the genetic determinants of individual response variability to PCSK9 pharmacological inhibition is a critical issue [7].

In real-world clinical settings, PCSK9i have been shown to be less effective in reducing LDL-C levels in women compared to men [8,9], with the underlying mechanisms to be clarified [9]. A tentative explanation for this observation could lie in the different PCSK9 concentrations between sexes [10], since large-scale clinical studies involving the collection of blood samples for the centralized measurement of PCSK9 showed that women have higher circulating PCSK9 than men [11]. Proposed alternative explanations for the unusual LDL-C response to PCSK9i include higher Lp(a) concentrations, which more commonly occur in postmenopausal women [9,12]. Treatment with PCSK9i has been shown to reduce LDL-C and Lp(a) in a 2:1 ratio (LDL-C approximately 50–60%: Lp(a) ≈25–30%), and often in a discordant manner (e.g., in >30% of individuals undergoing treatment, Lp(a) and LDL-C do not fall concordantly) [13,14]. In these cases, according to Warden et al., the reduced LDL-C response could be accounted for by the higher proportion of reported LDL-C consisting of Lp(a) particles, which are not cleared efficiently by the LDL receptor [12]. Unfortunately, unlike LDL-C, sex-dependent differences in the Lp(a)-lowering effect driven by PCSK9 inhibition have never been investigated before, neither in controlled clinical trials nor in real-world settings. Then, the aim of this study was to evaluate whether there were significant sex x time interactions in Lp(a) and LDL-C response to treatment with the PCSK9i Evolocumab.

## 2. Methods

### 2.1. Study Design and Participants

This is a subanalysis of an ongoing prospective observational study, whose protocol was approved by the Ethics Committee of the University of Bologna (Code: LLD-RP2018). The study followed the Declaration of Helsinki and its amendments, and all individuals signed an informed consent to participate.

Data were pooled from hypercholesterolemic patients recruited at the Lipid Clinic of the S. Orsola-Malpighi University Hospital, Bologna, Italy. Enrolled individuals were eligible for treatment with PCSK9i according to the recommendations of the European Society of Cardiology (ESC) and the European Atherosclerosis Society (EAS) [15], as well as the criteria released by the Italian Regulatory Agency (AIFA) [16,17]. Additional inclusion criteria were ≥18 years of age and being on maximum tolerated oral lipid-lowering therapy (statin and ezetimibe or ezetimibe monotherapy) for ≥6 months before starting Evolocumab, with no planned dose change.

**Figure 1:**
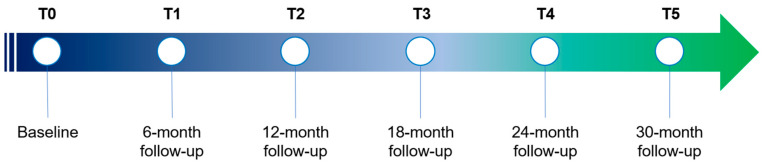
Study timeline.

Patients were clinically evaluated at baseline and every six months after starting Evolocumab (Figure 1). Individuals who had been on PCSK9i for less than 30 months and noncompliant patients were excluded from the analysis.

### 2.2. Assessments

#### 2.2.1. Clinical Data and Physical Assessments

Each patient’s personal history was evaluated paying particular attention to ASCVD, smoking habit and ongoing pharmacological treatments. Genetic screening for the presence of an FH-causing variant was done in case of clinical suspicion. Height and weight were measured to the nearest 0.1 cm and 0.1 kg, respectively, with patients standing erect with eyes directed straight, wearing light clothes and with bare feet. Body mass index (BMI) was calculated as body weight in kilograms, divided by height squared in meters (kg/m^2^) [18].

#### 2.2.2. Laboratory Analysis

Laboratory analyses were performed to investigate complete blood count (CBC), total cholesterol (TC), high-density lipoprotein cholesterol (HDL-C), triglycerides (TG), Lp(a), apolipoprotein B (Apo-B), apolipoprotein A1 (Apo-A1), fasting plasma glucose (FPG), serum uric acid (SUA), creatinine (Cr), total bilirubin and fractions, alanine transaminase (ALT), aspartate transaminase (AST), gamma-glutamyl transferase (gamma-GT), creatinine phosphokinase (CPK) and thyroid-stimulating hormone (TSH). Venous blood samples were obtained from each patient after overnight fasting. Lp(a) concentrations were measured using an immunoturbidimetric assay. LDL-C was calculated by the Friedewald formula [19]. The glomerular filtration rate (eGFR) was estimated by the Chronic Kidney Disease Epidemiology Collaboration (CKD-epi) equation [20].

### 2.3. Statistical Analysis

Data distribution was assessed by histograms, q-q plots and Shapiro–Wilk’s test. Continuous variables were summarized using arithmetic mean ± standard deviation, median and 1st/3rd quartiles and *n* (%). For two-group comparisons of descriptive clinical parameters, an independent two-sample *t*-test, Mann–Whitney U test and Pearson chi-squared test were used. To identify the main and interaction effects of sex and time points, nonparametric analysis of longitudinal data was applied for Lp(a) and LDL-C. Experimental results were summarized with Wald statistics, degrees of freedom and *p*-values. The Mann–Whitney U test was used for sex comparisons at each time point, separately. The Friedman test was used to compare the change over time in Lp(a) and LDL-C according to sex. Bonferroni and Nemenyi tests were applied for multiple comparisons. Area under the curve (AUC) values of Lp(a), LDL-C, percent change from baseline in Lp(a) and LDL-C were also calculated, and the median AUC values were compared by sex, using the Mann–Whitney U test. A *p*-value of <0.05 was considered statistically significant. All analyses were conducted using R 4.2.1 (www.r-project.org) software.

## 3. Results

According to the prespecified inclusion and exclusion criteria, we pooled data from 176 patients (Men: *n* = 93; Women: *n* = 83), who, in October 2022, had been treated with Evolocumab for at least 30 months. Baseline characteristics of the enrolled patients are reported in Table 1. The mean age and history of ASCVD were significantly higher in men than in women (*p*-value < 0.05). Following the classification of the American College of Cardiology (ACC) and the American Heart Association (AHA), the intensity of background statin therapy was reported as divided into 3 categories [21]. High-intensity statin use was defined as atorvastatin ≥ 40 mg or rosuvastatin ≥ 20 mg; moderate-intensity statin use was defined as atorvastatin ≤ 20 mg, rosuvastatin ≤ 10 mg, simvastatin ≥ 20 mg, pravastatin ≥ 40 mg, lovastatin ≥ 40 mg or fluvastatin 80 mg; low-intensity statin use was defined as simvastatin 10 mg, pravastatin ≤ 20 mg, lovastatin ≤ 20 mg or fluvastatin ≤ 40 mg. As reported in Table 1, the overall distribution of statin treatment was not different across men and women at the baseline (*p*-value > 0.05). The use of ezetimibe as background lipid-lowering therapy was higher in women than men (*p*-value < 0.05). The median values of TC, HDL-C, eGFR, ALT and gamma-GT were significantly higher in men than women (*p*-values < 0.05). In women, LDL-C, AST and CPK were higher (*p*-values < 0.05).

**Figure 2:**
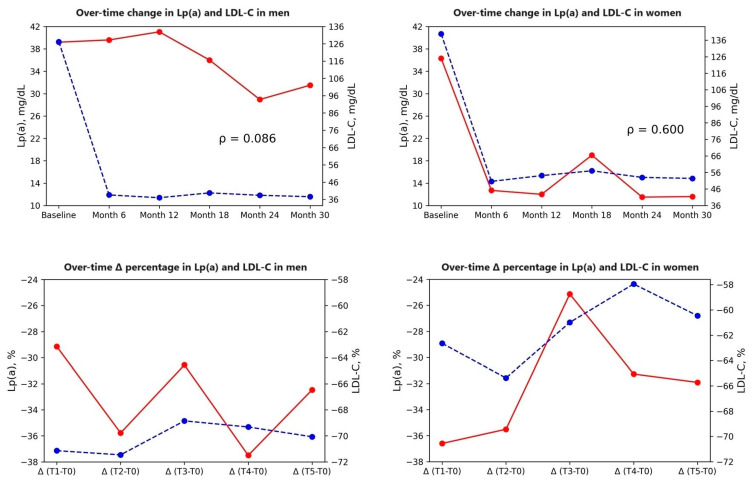
Longitudinal trends in LDL-C and Lp(a) plasma concentrations over time. LDL-C = Low-density lipoprotein cholesterol; Lp(a) = Lipoprotein(a). Dashed blue line: LDL-C; Solid red line: Lpa(a); ρ: Spearman correlation coefficient.

Absolute and percentage changes in LDL-C and Lp(a) for all patients and by sex are shown in Figure 2. A positive and moderate correlation was observed between the over-time change trends of Lp(a) and LDL-C measurements in women (ρ = 0.600). The relationship between these two measurement trends was observed to be quite weak in men (ρ = 0.086).

Nonparametric analysis of longitudinal data in factorial experiments for changes in plasma concentrations in Lp(a) and LDL-C is given in Table 2. The main effects and the interaction of the sex and time were found to be statistically significant in LDL-C (all *p*-values < 0.05). On the contrary, absolute changes in Lp(a) were influenced neither by sex nor by time (all *p*-value > 0.05) (Table 2).

Absolute values of Lp(a) plasma concentrations significantly decreased in the entire cohort (*p*-value < 0.001), without any difference among sex over time (*p*-value < 0.001 in men and *p*-value = 0.002 in women) (Table 3). No sex-related significant differences were detected at any time point (*p*-value > 0.05). Moreover, no statistically significant difference was found in the percentage changes between sexes (*p*-value > 0.05) (Table 3).

Absolute plasma concentrations of LDL-C significantly decreased over time, in the entire cohort (*p*-value < 0.001) and by sex (*p*-value < 0.001 in men and women) (Table 4). LDL-C concentrations remained significantly higher in women than men at each time point (*p*-values < 0.05 always); similarly, the AUC was higher in women than men (*p*-value = 0.017). LDL-C percentage significantly decreased more in men than in women at each time point (*p*-value < 0.01 always) (Table 4).

Treatment with the PCSK9i was well tolerated. No serious adverse event was registered during the follow-up.

## 4. Discussion

Over the last 30 years, there was an overall declining trend in age-standardized disability-adjusted life years (DALY) rate as regards ASCVD, with larger declines among women compared to men [22]. Today, the identification of sex-related differences in determinants of individual CV risk continues to receive considerable attention from the scientific community, to plan and implement prevention policies and programs, also regarding the early diagnosis and management of dyslipidemia [23].

The controversy about whether women benefit to the same extent as men from lipid-lowering treatment is mainly attributable to a relative lack of information about the effects on women from individual clinical trials [24]. This bias is secondarily due to sampling errors that led to the enrollment disparity difference between the proportion of women with prevalent ASCVD and the proportion of women enrolled in the studies [25].

Early-phase drug investigations have historically excluded women of childbearing age due to physiological hormonal fluctuations and concerns of safety for the mother and fetus if the woman had become pregnant after enrollment [26]. Women are also well known to develop CHD on average 10 years later than men, being more likely to be excluded due to age requirements from clinical studies enrolling individuals with ASCVD [27].

In addition to improving enrollment, another critical issue is to develop strategies to foster the retention of women participants [28], since women are more likely to withdraw consent from the trials and discontinue study drugs compared to men [29].

Available data are conflicting about the existence of long-term differences between women and men in response to PCSK9i Evolocumab. It has been assumed that a role could be played by Lp(a), whose plasmatic concentrations are affected by estrogen fluctuations [30,31]. This assumption has yet to be proven, and new data on the relationship between changes in Lp(a) and ASCVD risk reduction in women will come from the ongoing clinical trials testing the emergent Lp(a)-lowering drugs.

The siRNA agents (olpasiran, LY3819469 and SLN360) and the second-generation antisense oligopeptide pelacarsen are being developed to specifically interfere with Lp(a) synthesis in the liver by blocking the translation of apo(a) messenger RNA (mRNA) in apo(a) [32], and in the next years, the phase III pivotal CV outcome trials (CVOT)—Lp(a)HORIZON and OCEAN(a)—will definitively clarify whether lowering Lp(a) translates into improved ASCVD outcomes. However, according to the findings of a recently published dose–response meta-analysis providing a comprehensive overview of the association between circulating Lp(a) and all-cause and cause-specific mortality, the risk of death from ASCVD increases by 31% for each 50 mg/dL rise in Lp(a) plasma levels [33]. Then, in the absence of treatment options currently available for the effective management of patients with high Lp(a) levels [34], the Lp(a)-lowering effect driven by PCSK9 inhibition is particularly interesting.

The FLOREY (Effects on Lipoprotein Metabolism From PCSK9 Inhibition Utilizing a Monoclonal Antibody) Study firstly suggested that Evolocumab reduces Lp(a) through the inhibition of the synthesis of apo(a) and the upregulation of the LDLR activity [35]. A tentative explanation could be that Lp(a) can compete more favorably for the LDLR when LDL-C levels in plasma are very low [36]. However, if PCSK9 inhibition lowers Lp(a) exclusively through LDLR-mediated clearance, the Lp(a) response would likely be proportional to the LDL-C response, and this does not happen [14].

In our cohort of outpatients, there were no remarkable sex-related significant differences in Lp(a) response to Evolocumab. However, plasma concentrations of LDL-C significantly decreased over 2.5 years in the entire cohort and by sex, with greater improvements in men compared to women. The sex x time interaction was statistically significant in LDL-C, while absolute changes in Lp(a) were not influenced by either sex or time. Then, our analysis answers an important and clinically relevant question that was left open by the pivotal studies and subsequent research [14]. Overall, these observations are essential to gain a better understanding of the relationship between LDL-C and Lp(a) lowering in response to Evolocumab, since our findings are unlikely to be explained by non-sex differences in baseline characteristics between men and women. In addition, sex differences in response to treatment with Evolocumab are likely to be driven by a sex-hormone-independent mechanism, since our cohort consisted of postmenopausal women with none of them being on hormone replacement therapy or anti-estrogen therapy for breast cancer. According to this hypothesis, it is possible that PCSK9 inhibition modifies the composition of gut microbiota by interfering with the bile acids excretion differently in men and women, whereas gut dysbiosis could increase PCSK9 expression. All these mechanisms could be influenced by sex, with a consequent impact on LDL-C plasma levels unlike Lp(a), and a different response to PCSK9 inhibition [37]. Furthermore, PCSK9 inhibitors could exert different effects on inflammation in men and women and, consequently, on LDL-C. However, unlike other lipid-lowering drugs, PCSK9 inhibitors have no or marginal impact on the circulating levels of high-sensitivity C-reactive protein (hs-CRP) [38]. In the FOURIER (Further Cardiovascular Outcomes Research with PCSK9 Inhibition in Subjects with Elevated Risk) study, there was a stepwise risk increment according to the values of hs-CRP: +9% (<1 mg/L), +10.8% (1–3 mg/L) and +13.1% (>3 mg/L) even in patients with extremely low levels of LDL-C; however, no subanalysis by sex was carried out on the relationship between hs-CRP levels and changes in Lp(a) levels in men and women [39]. The reason why LDL-C decreased more in men than women in response to treatment with Evolocumab while Lp(a) similarly changed is yet to be elucidated.

Of course, our study has some limitations that need to be acknowledged. Firstly, background oral lipid-lowering therapy was heterogeneous in the cohort, and patients received the maximum tolerated dose of statin and/or ezetimibe or no treatment, based on the tolerability threshold. However, it is well known that statins and ezetimibe have no impact on Lp(a) levels. Additional limitations are that PCSK9 levels and apolipoprotein(a) (apo(a)) isoform size were not assessed. For this reason, the association between PCSK9 and LDL-C or Lp(a) cannot be investigated in this study. Moreover, we cannot establish whether the relative expression of apo(a) isoforms changes after the Lp(a) levels are lowered using Evolocumab. It is not even possible to ascertain that the size of the apo(a) is an independent determinant of the treatment response, even if one published study suggests that each additional kringle domain is with a 3% additional reduction in Lp(a) [40].

During follow-up, no major CV event or serious adverse event was registered, probably because all modifiable CV risk factors were strictly monitored throughout the observation period and optimized. However, it should be noted that the selection criteria of the analysis required an adherence rate to treatment of 100%. This may have led to an underestimation of adverse events as adverse events occurrence is one of the factors that most influence treatment nonadherence and discontinuation. Finally, this study was performed in a single center, and this may have had an impact on the sample size. However, all patients were treated according to the national PCSK9i reimbursement criteria, so our results are representative of individuals using PCSK9i in Italy.

## 5. Conclusions

In conclusion, our data partially reinforce the presence of differences in response to treatment to PCSK9i between men and women and are essential to gain a better understanding of the relationship between LDL-C and Lp(a) lowering in response to PCSK9i. Further research will clarify whether these sex-related significant differences translate into a meaningful difference in the long-term risk of ASCVD.

## Figures and Tables

**Table 1:** Baseline characteristics of the patients enrolled in the study.

Characteristics	All Patients (*n =* 176)	Men (*n =* 93)	Women (*n =* 83)	*p*-Values
Age (years)	63.4 ± 10.1	63.4 ± 10.5	61.9 ± 10.3	0.012
History of ASCVD (*n*; %)	105 (59.7)	53 (57.0)	52 (62.7)	0.021
Type 2 Diabetes Mellitus (*n*; %)	21 (11.9)	12 (12.9)	9 (10.8)	0.409
Familial Hypercholesterolemia (*n*; %)	64 (36.6)	30 (32.3)	34 (41.5)	0.207
Hypertension (*n*; %)	117 (66.5)	58 (62.4)	59 (71.1)	0.308
Background lipid-lowering therapy				
Statin (*n*; %)	75 (42.6)	36 (38.7)	39 (47)	0.188
High-intensity dosage (*n*; %)	28 (38.4)	14 (33.3)	14 (45.2)	
Moderate-intensity dosage (*n*; %)	45 (61.6)	28 (66.7)	17 (54.8)	
Low-intensity dosage (*n*; %)	0 (0)	0 (0)	0 (0)	
Ezetimibe (*n*; %)	119 (67.6)	54 (58.1)	65 (78.3)	0.012
BMI (kg/m^2^)	27 ± 4	26.8 ± 4.4	27.4 ± 4.3	0.122
TC (mg/dL)	214 (190–251)	216 (190–261.5)	205.5 (190–242.5)	0.003
LDL-C (mg/dL)	132.2 (111.2–166.8)	131.6 (111.3–173.8)	137 (111.2–161.8)	0.050
HDL-C (mg/dL)	53.8 ± 12.6	58.7 ± 11.9	48.9 ± 10	<0.001
TG (mg/dL)	132 (93.5–179)	139 (87.5–191.5)	119 (98.5–162.8)	0.842
Lp(a) (mg/dL)	39.2 (11.9–107)	39.2 (9.7–110.7)	36.3 (12–95.7)	0.722
eGFR (mL/min)	80 ± 18.7	82.6 ± 19.9	80.8 ± 20.6	0.030
AST (U/L)	25 (21–30)	25 (21.3–30)	26 (20–30.5)	0.001
ALT (U/L)	24 (17–32.5)	25 (18–31)	24 (16.3–33.8)	<0.001
Gamma-GT (U/L)	24.5 (17–35.3)	25 (17–39)	24 (16.5–33)	<0.001
CPK (U/L)	140.5 (85.8–236.8)	126 (84–206)	156 (91.5–262.5)	<0.001

ALT = Alanine transaminase; ASCVD = Atherosclerotic cardiovascular disease; AST = Aspartate transaminase; BMI = Body mass index; CPK = Creatinine phosphokinase; eGFR = Estimated glomerular filtration rate; Gamma-GT = Gamma-glutamyl transferase; HDL-C = High-density lipoprotein cholesterol; LDL-C = Low-density lipoprotein cholesterol; Lp(a) = Lipoprotein(a); *n* = Number of patients; TC = Total cholesterol; TG = Triglycerides. Values are expressed as mean ± standard deviation, median (1st/3rd quartiles) and *n* (%).

**Table 2:** Nonparametric analysis of longitudinal data in factorial experiments for changes in plasma concentrations in Lp(a) and LDL-C.

Source of Variation	df	Wald	*p*-Values
Lp(a)			
Sex	1	2.839	0.092
Time	5	10.965	0.052
Time ^x^ Sex	5	2.869	0.720
LDL-C			
Sex	1	16.843	<0.001
Time	5	855.501	<0.001
Time ^x^ Sex	5	14.005	0.016

df = Degrees of freedom; LDL-C = Low-density lipoprotein cholesterol; Lp(a) = Lipoprotein(a).

**Table 3:** Between-sex changes in Lp(a) concentrations over time. Values are expressed as median (1st/3rd quartiles).

Time Points	All Patients (*n =* 176)	Men (*n =* 93)	Women (*n =* 83)	*p*-Values ^†^
Lp(a)				
Baseline (T0)	39.2 (11.9/107) ^a^	39.2 (9.7/110.7) ^ab^	36.3 (12/95.7) ^a^	0.722
6 Months (T1)	30.1 (6.4/79.7) ^b^	39.6 (8.4/101.9) ^ab^	12.7 (5.2/64.9) ^ab^	0.063
12 Months (T2)	24.3 (8.1/72.9) ^b^	41.1 (10.5/79.1) ^a^	12 (6.6/68.6) ^b^	0.054
18 Months (T3)	28.5 (8.6/74.3) ^b^	36 (10.8/91.7) ^b^	19 (5.9/65.4) ^ab^	0.091
24 Months (T4)	22.9 (6.4/83.7) ^b^	29 (6.9/97.5) ^b^	11.5 (5.9/64.8) ^b^	0.134
30 Months (T5)	27.6 (8.1/87.3) ^b^	31.5 (9.2/103.2) ^b^	11.6 (6.5/85.4) ^b^	0.176
*p*-value ^‡^	<0.001	<0.001	0.002	
AUC	1290.60 (316.50/3146.70)	2255.55 (489.15/3721.43)	1237.80 (1057.80/1932.60)	0.125
Delta %				
T1-T0	−33.62 (−43.56/−14.71)	−29.15 (−43.08/−16.34)	−36.60 (−45.34/−9.09)	0.865
T2-T0	−35.64 (−52.28/−13.71)	−35.78 (−52.36/−16.78)	−35.51 (−52.10/−8.68)	0.722
T3-T0	−29.22 (−45.04/−10.31)	−30.56 (−42.03/−15.17)	−25.13 (−46.53/0.89)	0.605
T4-T0	−36.36 (−61.08/−13.55)	−37.5 (−62.05/−14.41)	−31.27 (−52.69/−8.11)	0.681
T5-T0	−32.24 (−56.34/−17.89)	−32.47 (−54.73/−12.42)	−31.91 (−56.34/−18)	0.988

AUC = Area under the curve; Lp(a) = Lipoprotein(a); n: Number of patients. Delta % = 100 × ((Ti-T0)/T0). ^†^: between-group comparison; ^‡^: within-group comparisons. Different lowercase letters (^a,b^) in the same column indicate a statistically significant difference between the time points.

**Table 4:** Between-sex changes in LDL-C concentrations over time. Values are expressed as median (1st/3rd quartiles).

Time Points	All Patients (*n =* 176)	Men (*n =* 93)	Women (*n =* 83)	*p*-Values ^†^
Baseline (T0)	132.2 (111.2/166.8) ^a^	127.2 (107/156.2) ^a^	139.8 (113.9/177.7) ^a^	0.049
6 Months (T1)	45.4 (28.6/69.4) ^b^	38.6 (22.6/56.1) ^b^	50.6 (36.4/78.3) ^b^	0.002
12 Months (T2)	44.8 (27.4/72) ^b^	37 (24.6/56) ^b^	54.1 (33.6/93.8) ^b^	0.001
18 Months (T3)	42.7 (30.2/67.7) ^b^	39.8 (25.8/55.2) ^b^	57 (34.6/78.3) ^b^	0.001
24 Months (T4)	47.6 (31.3/69.1) ^b^	38.4 (24.7/60.9) ^b^	53 (40/87) ^b^	0.002
30 Months (T5)	44.3 (28.7/64.5) ^b^	37.6 (23.6/54) ^b^	52.4 (41.4/80) ^b^	<0.001
*p*-value ^‡^	<0.001	<0.001	<0.001	
AUC	1543.20 (1113.60/2245.65)	747.6 (228.9/1583.4)	1915.8 (1324.2/2623.2)	0.017
Delta %				
T1-T0	−67.8 (−76.43/−55.46)	−71.14 (−81.26/−60.54)	−62.65 (−71.44/−51.3)	0.004
T2-T0	−68.15 (−77.29/−50.75)	−71.46 (−79.41/−59.06)	−65.38 (−75.82/−37.93)	0.007
T3-T0	−66.6 (−75.25/−55.1)	−68.85 (−78.02/−61.84)	−60.99 (−73.35/−45.88)	0.006
T4-T0	−65.44 (−76.02/−49.91)	−69.32 (−79.37/−53.25)	−57.95 (−70.02/−49.1)	0.010
T5-T0	−65.77 (−75.5/−53.77)	−70.07 (−79.96/−58.23)	−60.46 (−72.18/−44.88)	0.006

AUC = Area under the curve; LDL-C = Low-density lipoprotein cholesterol; n: Number of patients. Delta % = 100 × ((T1-T0)/T0). ^†^: between-group comparison; ^‡^: within-group comparisons. Significant *p*-values are shown in bold. Different lowercase letters (^a,b^) in the same column indicate a statistically significant difference between time points.

## Data Availability

Data supporting the findings of this analysis are available from the University of Bologna. Data are available from the authors with the permission of the University of Bologna.
